# On the Preparation of Zinc Ointment

**Published:** 1855-08

**Authors:** David Kemp


On the Preparation of Zinc Ointment. By David Kemp.—(Pharm. Journal, Dec. 1854, p. 280.

The softness and want of durability of zinc ointment, as prepared according to the London Pharmacopœia, lead Mr. Kemp to propose the following formula:—℞ Axungiæ opt., olei oliæ purif., ana ℥x; ceræ albæ, cetacei, oxidi zinci, ana ℥v. Melt in a water bath, strain into a warm mortar, and just before cooling add the zinc, and continue stirring very briskly till quite cold. If the temperature be too high when the zinc is added, the color is impaired. He has never observed this ointment, which is of good consistence, to show any peculiar tendency to become rancid, or to require any unusual means to be employed for its preservation. *Ibid.*

